# Examining Parent Mood, Feeding Context, and Feeding Goals as Predictors of Feeding Practices Used by Parents of Preschool Children With Avid Eating Behavior: Protocol for an Ecological Momentary Assessment Study

**DOI:** 10.2196/55193

**Published:** 2024-03-19

**Authors:** Katie Edwards, Helen Croker, Claire Farrow, Emma Haycraft, Moritz Herle, Clare Llewellyn, Abigail Pickard, Jacqueline Blissett

**Affiliations:** 1 School of Psychology and Institute of Health and Neurodevelopment Aston University Birmingham United Kingdom; 2 World Cancer Research Fund International London United Kingdom; 3 School of Sport, Exercise and Health Sciences Loughborough University Loughborough United Kingdom; 4 Social, Genetic & Developmental Psychiatry Centre Institute of Psychiatry, Psychology & Neuroscience King’s College London London United Kingdom; 5 Research Department of Behavioural Science and Health Institute of Epidemiology and Health Care University College London London United Kingdom

**Keywords:** ecological momentary assessment, avid eating, children’s eating behavior, parental feeding practices, feeding behaviour, parent, children, eating behaviour, obesity, environmental factors, observational study, feeding, United Kingdom

## Abstract

**Background:**

An avid eating behavior profile is characterized by a greater interest in food and a tendency to overeat in response to negative emotions. Parents use specific strategies to manage feeding interactions with children with avid eating behavior. While momentary and contextual factors, such as parental mood, have been found to influence parental feeding practices, there is a lack of research examining parents’ daily experiences of feeding children with avid eating behavior. Examining this is important because parental feeding practices are key levers in tailored interventions to support children’s healthy eating behavior.

**Objective:**

We aim to describe the ecological momentary assessment methods and procedures used in the APPETItE (Appetite in Preschoolers: Producing Evidence for Tailoring Interventions Effectively) project, which aims to examine how variation in parental mood, feeding goals, and the context of eating occasions affect the parental feeding practices used to manage feeding interactions with children with an avid eating behavior profile.

**Methods:**

Participants are primary caregivers from the APPETItE cohort who have a preschool-age child (aged 3-5 years) with an avid eating behavior profile. Caregivers complete a 10-day ecological momentary assessment period using signal- and event-contingent surveys to examine (1) mood and stress, (2) parental feeding goals, and (3) contextual factors as predictors of parental feeding practices.

**Results:**

Recruitment and data collection began in October 2023 and is expected to be completed by spring 2024. The data have a 3-level structure: repeated measurements (level 1) nested within days (level 2) nested within an individual (level 3). Thus, lag-dependent models will be conducted to test the main hypotheses.

**Conclusions:**

The findings from this study will provide an understanding of caregivers’ daily experiences of feeding preschool children with avid eating behavior, who are at greater risk for the development of obesity. Understanding the predictors of feeding practices at the moment they occur, and across various contexts, will inform the development of tailored resources to support caregivers in managing children’s avid eating behavior.

**International Registered Report Identifier (IRRID):**

DERR1-10.2196/55193

## Introduction

Individuals differ in their susceptibility to obesity due to complex interactions between genetic and environmental factors that contribute to the development of eating behavior [1]. One population who are at greater risk of developing obesity is children with more avid appetites. Avid eating behavior is characterized by higher enjoyment of food, greater responsiveness to food cues, a tendency to overeat in response to negative emotions, faster eating, less sensitivity to satiety cues, and lower levels of food fussiness [2,3]. We have established that around 1 in 5 children aged 3-5 years in the United Kingdom show an avid eating behavior profile [3]. The findings from prospective research have shown that avid eating behaviors are positively associated with children’s adiposity [4]. Thus, children with avid eating behavior may be at greater risk of developing obesity. Given the immediate and long-term negative health consequences associated with childhood obesity [5], it is vital to identify strategies across time and contexts to support the development of healthy eating behavior for children with avid eating behavior profiles.

One powerful and modifiable influence on the development of children’s eating behavior is the feeding practices used by parents or caregivers which directly influence what, when, and how much food children consume [6,7]. We have demonstrated that parents of children aged 3-5 years with an avid eating behavior profile use more restriction of food and greater use of food for emotion regulation than those children without avid eating behavior [3]. However, this analysis, like much research examining parental feeding practices, has relied on static, self-report measures that do not capture the intraindividual variability in parent feeding behavior across time and context. Further, 1 methodology that has been used more recently to capture variations in factors that influence feeding behavior is ecological momentary assessment (EMA). Research using EMA has extended our understanding of parent-child feeding interactions, demonstrating that parental feeding practices differ across time and contexts (eg, the type of eating occasion) [8,9]. This suggests that the use of specific parental feeding practices is situation-dependent and highlights the complexity of parent-child feeding relationships. While the use of momentary parental feeding practices remains to be examined in children with avid eating behavior, findings from qualitative research suggest that parental feeding practices used to manage children’s avid eating behavior also vary by context, such as the type and location of the eating occasion [2]. For example, parents of children with an avid eating behavior profile reported greater use of controlling feeding practices at snack times, compared to mealtimes. Therefore, since feeding practices to manage children’s avid eating behavior appear situation-dependent, research using momentary observation (eg, EMA) is needed to examine the effect of contextual factors on feeding interactions with children with avid eating behavior.

Other key influences on parental feeding practices include parental mood and stress [10]. EMA research has demonstrated that fluctuations in parental mood and stress throughout the day influence the subsequent use of parental feeding practices. For example, higher maternal stress and depressed mood earlier in the day have been found to predict feeding practices (eg, greater pressure to eat), and the type of food served in the evening (eg, fewer homemade meals) [11-13]. Furthermore, qualitative research has shown that parents use specific feeding strategies to manage challenging feeding interactions with children with avid eating behavior [2]. For example, parents described using indulgent feeding strategies, such as emotional feeding and food as a reward, when parenting energy is low. Given this, it is possible that momentary changes in parental mood and stress could influence the use of subsequent feeding practices with children with avid eating behavior. This remains to be examined but is critical for the development of effective support targeting feeding practices of parents of children with avid eating behavior. If mood and stress are key predictors of the use of less adaptive strategies, our intervention must provide support tailored for the management of those experiences.

Parental feeding goals have also been shown to influence feeding interactions [14]. Parental feeding goals may predict feeding practices, for example, parents may use greater restriction of less healthy foods when their feeding goal is health-related. Previous research has specifically focused on mealtime feeding goals [14], however, it is also important to investigate parental feeding goals during snack times, to determine whether feeding goals vary by time and context. Examining this is particularly important because parents of children with avid eating behavior have described their children as frequently requesting snacks [2]. Additionally, it is possible that parental mood throughout the day could influence subsequent feeding goals. For example, parents might aim to avoid stress and conflict at an evening meal after experiencing high stress throughout the day. However, whether fluctuations in parental mood throughout the day influence subsequent feeding goals remains to be examined.

In summary, EMA research has extended our understanding of parent-child feeding interactions, demonstrating that parental feeding practices are situation-dependent and are influenced by momentary factors, such as context and parental mood [8,11-13]. Feeding children with avid eating behavior is challenging [2], however, there is a lack of research examining parents’ daily experiences. Thus, examining feeding interactions as they occur in real time and contexts using EMA will provide insight into the parents’ experiences of feeding their child with avid eating behavior. Hence, this protocol aims to provide a detailed overview of the EMA methods and procedures used in the APPETItE (Appetite in Preschoolers: Producing Evidence for Tailoring Interventions Effectively) project. This study aims to examine how variation in parental mood, feeding goals, and the context of eating occasions affects the parental feeding practices used to manage feeding interactions with children with an avid eating behavior profile.

## Methods

### Study Design and Participants

This observational study is part of a larger program of research (APPETItE project) which examines feeding and eating in preschool children with avid eating behavior, to inform future intervention design and efficacy. The APPETItE project has identified an avid eating behavior profile in preschool children using latent profile analysis [3]. Primary caregivers (N=200) of a child aged 3-5 years who was identified as having an avid eating behavior profile in this initial study [3] are invited to participate in this EMA study. Given the novelty of this research, a reliable power calculation could not be conducted. Thus, based on previous research [11] we aimed to invite 200 parents to participate to account for attrition and to provide sufficient data to examine within- and between-subject effects. Eligibility criteria include English-speaking primary caregivers from the United Kingdom who are responsible for feeding their child for more than half the time when their child is at home. Caregivers whose child is autistic, has severe learning disabilities, or a chronic illness that directly influences their dietary requirements and eating habits are not eligible to participate.

### Ethical Considerations

Ethical approval was provided by the Aston University Health and Life Sciences Research Ethics Committee (HLS21003). All participants are asked to provide informed consent.

### Recruitment

Eligible primary caregivers are invited to participate by email. After registering their interest to participate, caregivers receive an email including details of how to download the mobile app, and how to complete the baseline questionnaire. Following completion of the baseline questionnaire, caregivers receive an email including details about how to complete the survey period, along with an information video and leaflet. If caregivers experience any technical difficulties, have any questions, or want to go through this study’s procedures, they can contact the research team by email or arrange a video call.

### Data Collection Procedures

#### Overview

Caregivers complete a baseline questionnaire, 10 days of EMA, and an end-of-study questionnaire. Participation in this study is remote; surveys are administered through a mobile smartphone app, which is downloaded directly to caregivers’ personal smartphones. Caregivers who do not own a smartphone can request one from the research team to use for this study’s period. This study, including a list of all the items used, was preregistered on the Open Science Framework [15].

#### Baseline Questionnaire

The baseline questionnaire gathers information about caregivers’ food security and general mood (anxiety, depression, stress, and well-being). Caregivers are also asked to provide their home postcode as a measure of Index of Multiple Deprivation. The Index of Multiple Deprivation decile scores are calculated by ranking residential areas in England into 10 equal groups, with scores ranging from 1 (indicating the most deprived 10% of residential areas) to 10 (indicating the least deprived 20%-30% of residential areas) [16]. Caregivers’ food security is measured using the Short Form of the Household Food Security Scale [17]. Responses are summed and categorized as 0-1 = high or marginal food security, 2-4 = low food security, and 5-6 = very low food security.

Additionally, caregivers are asked to complete 3 questionnaires to assess their general mood. The Hospital Anxiety and Depression Scale [18] measures caregivers’ anxiety (7 items, eg, “I feel tense or wound up”) and depression (7 items, eg, "I still enjoy the things I used to enjoy"). Responses are on a 4-point Likert scale from 0 (eg, “not at all”) to 3 (eg, “most of the time”). The Hospital Anxiety and Depression Scale has been found to have good reliability and validity [19]. The Perceived Stress Scale [20] measures caregivers’ baseline stress across 10 items (eg, “in the last month, how often have you felt nervous and stressed?”), with scores from 0 (“never”) to 4 (“very often”). The Perceived Stress Scale is a valid and reliable measure [21]. Finally, the World Health Organization Well-Being Index [22] assesses caregivers’ well-being across 5 items (eg, “I have felt calm and relaxed”), with scores from 0 (“at no time”) to 5 (“all of the time”). The World Health Organization Well-Being Index has been found to have good reliability and validity [22].

Caregivers also provide information about how much time they usually spend with their child on a weekday and weekend, upcoming periods where their typical eating routine is altered (eg, holidays and feasting or fasting for religious reasons), or periods where they are not with their child (eg, shared custody). This information is gathered so that the EMA period can be adjusted to suit participants (eg, delaying the start date of the EMA period).

#### Ten-Day EMA Period

##### Overview

Caregivers complete 10 days of EMA surveys. While 7 complete days are required for this study, caregivers are asked to complete 10 consecutive days of EMA to allow flexibility for some incomplete days (eg, a sensitization period) [23]. The EMA period examines parents’ mood, feeding goals, feeding practices, and contextual factors using three sampling schemes: (1) signal-contingent surveys (morning and mood surveys), (2) event-contingent surveys (food surveys), and (3) end-of-day surveys (Table 1). Surveys take less than 5 minutes for caregivers to complete.

**Table 1:** Primary variables and covariates assessed during the 10-day EMA^a^ period.

Survey (delivery) and variables			Example item
**Mood survey (notification)**			
	Feeding goals^b^	To give my child food that is nutritious	
	Mood	I feel annoyed	
	Stress	I feel tense	
	Context	Who am I with?	
**Food survey (self-initiated)**			
	Feeding practices	Did you have to make sure your child did not eat too much food?	
	Feeding goals	I didn’t want to give in to my child, even if this caused an argument	
	Context	Where did your child ask for food?	
**End-of-day survey (notification)**			
	Mood	How stressful was your day?	
	Feeding practices	Today, how often did you have to limit your child’s eating of snack foods?	
	Children’s eating behavior	How satisfied are you with how your child ate today?	

^a^EMA: ecological momentary assessment.

^b^Examined at the morning survey only.

##### Signal-Contingent Surveys

Each day, caregivers are sent four signal-contingent surveys at semirandom times within one of four 120-minute blocks: 7 AM to 9 AM (morning survey); 10 AM to noon; 1 PM to 3 PM; and 4 PM to 6 PM. Each block is separated by 60 minutes, to avoid overlap between surveys. To accommodate different routines, the notification window for morning surveys can be adjusted between 5 AM and 9 AM. When a survey is ready to complete, caregivers receive a notification on their phone. A reminder notification is sent 15 minutes after the initial notification, if the survey has not been completed, to ensure that responses are momentary [24]. From the first notification, caregivers have 60 minutes to complete notification surveys before the link expires.

Signal-contingent surveys examine caregivers’ mood (positive and negative affect), stress, and the context in which the survey is completed. Items measuring positive and negative affect are adapted from the Positive and Negative Affect Schedule [25] and have been used extensively in EMA research [26]. Items measuring parents’ stress are adapted from the Perceived Stress Scale [20] for use in EMA research [23,27]. All responses are made on a 5-point Likert scale from 1 (“not at all”) to 5 (“extremely”). Surveys also examine the context in which caregivers complete the survey, using questions adapted from the PsyMate (Department of Psychiatry and Psychology at Maastricht University) standard assessment protocol [28]. To determine whether feeding goals vary throughout the day, morning surveys examine parental feeding goals. Questions are adapted from the Family Mealtime Goals Questionnaire [14] to assess caregivers’ health-related goals and stress and conflict avoidance goals. Feeding goals are not examined in other mood surveys.

##### Event-Contingent Surveys

Event-contingent (food) surveys are self-initiated by caregivers each time their child asks for or consumes food when their caregiver is present. For example, eating occasions when children are at preschool will not be reported. Caregivers have previously reported that children with avid eating behavior frequently ask for food [2], thus, caregivers are asked to report each time their child requests food, even if their child is not given food (eg, not allowing children a snack before dinner). Food surveys involve (1) “food consumed” surveys and (2) “food request” surveys. While the same broad domains are assessed, questions are adapted to suit the type of feeding situation. For example, “food consumed” surveys ask caregivers about their goals for the eating occasion (“what was your aim for this meal or snack time?”), whereas “food request” surveys ask caregivers about their goal for saying no (“what was your aim for saying no to your child when they asked for food?”). Caregivers are directed to complete a “food consumed” survey if they report that their child has eaten or to complete a “food request survey” if they report their child asked them for food but has not eaten.

Food surveys examine parental feeding practices across several domains: structure-related, autonomy support, coercive control, and indulgent feeding practices. Indulgent feeding practices are only measured in “food consumed” surveys because questions relate to occasions where children consume food. Questions are adapted from the Real-Time Parent Feeding Practices measurement tool [29]. Food surveys also examine parental feeding goals, including health-related goals, and stress and conflict avoidance goals. Questions are adapted from The Family Mealtime Goals Questionnaire [14] to suit the EMA format. For example, responses were changed from a 5-point Likert scale to “yes” or “no.” A “not applicable” option was also included for questions about parental feeding practices. When assessing parents’ main feeding goal, the “other” response option can be selected for parents to specify their own feeding goal. Questions examining the context of eating occasions and requests for food are adapted from the EMA component of the Family Matters study [23].

Completing event-based surveys relies on self-initiation by caregivers, thus, there may be occasions when caregivers forget to report children’s food requests or consumption. To ensure these feeding interactions are captured, each EMA survey ends with the option to report an occasion where children have asked for, or consumed food, that caregivers have not previously reported, as done in other EMA research [23,30].

##### End-of-Day Surveys

Each day, caregivers are asked to complete an end-of-day survey to provide a summary of how their day has been. Caregivers receive a semirandom notification between 8 PM and 10 PM when the end-of-day survey is ready to complete. Caregivers can adjust the survey window between 7 PM and midnight, to suit their evening routines. A reminder notification is administered 15 minutes after the first notification if caregivers have not yet completed the survey. End-of-day surveys are available for 60 minutes after the initial notification, before expiring.

End-of-day surveys include general questions about parental mood and feeding practices, the amount of time parents spent with their child, children’s eating behavior, and a feasibility question [23]. Questions also assess the number of meals and snacks children consumed and requested throughout the day, to help determine parents’ compliance with completing food surveys.

#### Feasibility and Usability

After the 10-day EMA period, caregivers complete a short questionnaire to assess the feasibility and usability of completing the EMA period. Questions are adapted from the PsyMate standardized protocol [31]. Examining the feasibility and usability of completing EMA research is important in this sample since feeding interactions with children with avid eating behavior can be challenging [2].

### Incentives

Caregivers receive a £100 (approximately US $126) shopping voucher if at least 8 days of surveys are complete. Based on previous research [29], the criteria for 1 complete day of EMA includes the completion of 2 signal-initiated (random) surveys and 1 event (food) survey. This accommodates for a variation in routines (eg, families where the child consumes most of their food in childcare settings), but also provides sufficient data to analyze within-day effects. All caregivers who complete 10 days of EMA are entered into a prize draw to win an additional £100 (approximately US $126) shopping voucher. If participants withdraw prematurely from this study, their time is reimbursed on a pro rata basis of £10 (approximately US $12.60) per complete day. The digital shopping vouchers are emailed to participants after taking part.

### Pilot Study

Study procedures were piloted with 25 primary caregivers of children aged 3-6 years with high food approach tendencies, to determine the feasibility and usability of this study’s processes. All caregivers provided feedback about their participation in this study which informed researchers of the adjustments needed (eg, increasing the time surveys are available) and technical difficulties.

## Results

### Overview

Recruitment and collection of data began in October 2023. At the time of this paper’s submission, data collection is ongoing and is anticipated until spring 2024. This study hypothesizes that parental mood, feeding goals, and the context of eating occasions (eg, meal setting) will predict the feeding practices that parents use to manage children’s avid eating behavior. Specifically, analyses will test the following hypotheses: (1) higher momentary stress, negative affect, and goals of reducing mealtime chaos will predict use of coercive and instrumental feeding practices at the subsequent eating occasion; (2) parental feeding goals will change throughout the day in response to parental mood, feeding goals, and mealtime context; (3) momentary mood and context will be associated with parental feeding goals; (4) parents will be more likely to report coercive and instrumental feeding practices when in public and when they report the atmosphere as tense or stressful; and (5) parental low mood, high stress, increased requests for food will predict changes in feeding goals.

### Statistical Analysis Plan

SPSS (IBM Corp) will be used for data cleaning and descriptive analyses, and R (R Foundation for Statistical Computing) will be used for main analyses. Skewness and kurtosis will be evaluated before data analysis, with relevant transformations administered if data violate the assumptions of the model. The data have a 3-level structure: repeated measurements (level 1) nested within days (level 2) and nested within an individual (level 3). Thus, lag-dependent models will be used to test our main hypotheses. More details of the analytic plan are on the Open Science Framework [15].

## Discussion

### Principal Findings

This will be the first study to examine how fluctuations in parental mood, feeding goals, and the context of eating occasions affect the parental feeding practices used with children with avid eating behavior. Determining momentary and contextual factors that influence parental feeding behavior is essential for better understanding caregivers’ daily feeding experiences. This is particularly important for caregivers of children with avid eating behavior since eating occasions have been reported as frequent and challenging [2].

However, experiencing frequent and challenging feeding occasions could negatively impact caregivers’ engagement with EMA. For example, caregivers would need to complete many food surveys, making this study more burdensome. In addition, time constraints have been reported to impact feeding decisions [32]; thus, caregivers of preschool children who are time-poor may struggle to engage with the EMA period. Hence, this study will provide an important understanding of the feasibility of conducting EMA research with a diverse sample of caregivers of preschool children with challenging eating behavior.

### Strengths and Limitations

This use study uses a novel methodology to gather a large amount of data about parental behavior across multiple time points and contexts. This novel approach will further our understanding of parental feeding practices, which has been predominantly based on static self-report measures, by capturing the intraindividual variability in feeding behavior across time and context. However, this study has several limitations. First, the use of a mobile app in this study relies on participants having good technological literacy, which may exclude participants from underrepresented communities. To improve the accessibility of this study, researchers from the APPETItE team can be contacted by email, web-based video chat, or phone to discuss and troubleshoot issues. Second, the limited number of items used in this study may not fully reflect parents’ experiences. However, given the large number of surveys, each survey must be short enough to reduce participant burden, while gathering sufficient information about parent behavior. Finally, the 10-day survey period may be particularly intensive for parents of preschool children with avid eating behavior given the expected frequent requests for food. This could potentially result in high attrition and data loss, and limit the findings to parents with the resources to complete multiple surveys. Given this, and the novelty of EMA research in this area, it is important to examine the feasibility of using this methodology with parents who may be experiencing feeding challenges with their child.

### Conclusion

Overall, this novel study will provide momentary evidence about caregivers’ daily experiences of feeding preschool children with avid eating behavior. The findings will contribute to the APPETItE project which aims to produce tailored guidelines to help parents nurture the development of children’s healthy eating behavior, particularly for children who are at greater risk for the development of obesity.

## Abbreviations

APPETItE: Appetite in Preschoolers: Producing Evidence for Tailoring Interventions Effectively
EMA: ecological momentary assessment

## Data Availability

The data sets generated and analyzed during this study will be available from the corresponding author upon reasonable request.
